# Digital Innovation in Multiple Sclerosis Management

**DOI:** 10.3390/brainsci12010040

**Published:** 2021-12-29

**Authors:** Tjalf Ziemssen, Rocco Haase

**Affiliations:** Multiple Sclerosis Center Dresden, Center of Clinical Neuroscience, Department of Neurology, University Clinic Carl-Gustav Carus, Dresden University of Technology, Fetscherstrasse 74, 01307 Dresden, Germany; rocco.haase@uniklinikum-dresden.de

The development of digital applications and remote communication technologies for people with multiple sclerosis (pwMS) has increased rapidly in recent years. eHealth apps have been shown to improve outcomes and facilitate access to care, disease information, and support. On the patient side, pwMS facing a disease onset in their early adulthood are often seen as the ideal target group for new trends in digital healthcare because of their demand for a more personalized and tailored disease management that results from the complexity and heterogeneity of multiple sclerosis (MS). For healthcare professionals (HCPs) treating MS, eHealth technologies can facilitate clinical disability assessment; analysis of laboratory and imaging data; and the remote monitoring of patient symptoms, adverse events, and outcomes. They can enable time optimization and more timely intervention than is possible with scheduled in-person visits.

This Special Issue addresses screening and assessment; disease surveillance; self-management, treatment, and rehabilitation; and counseling and education using digital tools for MS. In particular, we collected research that paints a more detailed picture of pwMS and their practitioners as eHealth users, and research that shows progress in measuring and diagnosing MS as well in the treatment and rehabilitation through digital innovations.

Haase et al. continued their investigation of active stakeholders in the process of digital MS management in a multi-survey study [1]. They took a close look at the attitudes, needs, and behaviors of pwMS, as well as at their relatives and caregivers regarding electronically assisted disease management. There was broad and robust enthusiasm among various subgroups. For pwMS, the focus was on eHealth services that connect information already collected and make it easily accessible and understandable. HCP preferred digital solutions that provided aid in the preparation of future visits and adherence. 

During the COVID-19 pandemic, remote medicine and education has become part of the new normal for patients and clinicians, introducing innovative care delivery models that are likely to endure. The master’s program “Multiple Sclerosis Management”, a digital program for HCPs at Dresden International University, was evaluated by Voigt et al., confirming feasibility and acceptability of a highly specialized study program that focuses solely on the management of one disease and delivers best-practice knowledge in digital form in 90% of lessons over a course of two years [2].

Due to the heterogeneous phenotype of the disease and large time intervals between neurologic examinations, measuring MS remains a complex task. Digital innovations may provide a solution to the problem of how we can avoid missing disease activity. Mäcken et al. developed a digital solution for one of the most common symptoms of MS, fatigue [3]. They included patient-reported outcomes, cognitive tests, and sensor data in a smartphone app applying a transtheoretical model of health behavior change that manifests in a training course for pwMS facing fatigue. Both patients and HCPs may benefit from objective fatigue assessments that can be easily administered by the patients themselves. In another article, van der Walt et al. explored the development pathway for a software as a medical device in MS, leveraging lessons learned from the development of Floodlight™ MS, an evolving app for MS functional assessment [4]. The strength of this solution is the integration of hand motor testing, gait tests, as well as cognitive and affective assessment under highest regulatory standards. With a special focus on gait testing, Trentzsch et al. developed a digitally assisted measurement system that uses accelerometers and multiple algorithms to assess the distance of a 2-min walk test [5]. The benefit of such a system is the simultaneous realization of standardization as well as objectification of measurement, which can be applied independently of human resources.

Towards a holistic approach to digital measurement of pwMS, Dillenseger et al. provided a comprehensive introduction into digital biomarkers in MS [6]. Digital biomarkers have the potential to close temporal gaps in diagnostics, to capture problem areas not addressed in clinical practice, to compile separate data sources in a timely manner, and thus to pave the way to personalized medicine at the pace of a clinical decision conversation. Therefore, digital biomarkers may include data from various sensors, tablets, medical devices. as well as video- and audio-based data and lead directly to the use of complex (big data) analyses that are largely based on machine learning approaches. A digital twin is a clinically useful representation of the knowledge gained in this way [7], which allows the treatment concept to be more data-driven at the individual longitudinal level as well as at the normative population level.

Cloosterman et al. presented a study on the potential impact of such a digital biomarker approach by assessing costs and benefits of the MS Sherpa app and online portal [8]. In this case, they performed an early health technology assessment that simulates the added value of digital biomarker-based eHealth interventions to the standard MS care path. Cost-effectiveness was demonstrated in all simulated scenarios, suggesting that digital biomarkers can be a valuable addition to routine clinical practice even with a small reduction in progression. With the software-assisted assessment of brain magnet resonance imaging (MRI) data, Sima et al. evaluated another digital biomarker and its impact on therapeutic decision-making [9]. A simulation on the effectiveness of an MRI-triggered switch of the disease-modifying therapy resulted in an increase in quality-adjusted life years and reduced societal costs due to MS.

A switch of treatments may be one result of the use of innovative digital technologies to treat MS. Beyond this, however, there are more facets and constellations in which eHealth can offer a contribution to improved treatment. In their review, Scholz et al. discussed the different types of interventions, standards, and advantages of quality eHealth approaches for pwMS [10]. They laid out several MS-specific use cases, such as single-use, social, integrated, and complex eHealth solutions, and collected factors of success for eHealth interventions in MS. In a second review, Bonnechère et al. focused on the existing clinical evidence of mobile health (mHealth) technologies in the rehabilitation and self-assessment of pwMS [11]. They reported small benefits of mHealth for cognitive functioning and moderate benefits for fatigue. For quality of life, further evidence on the level of activity and motor function was requested.

To promote an easy-to-access platform for interoperable data sharing and disease management across several HCPs, Lang et al. developed a CE-certified mobile application that provides risk management plans of current disease modifying therapies for MS [12]. Its use is not restricted to MS but already 3000 pwMS have used this integrative system that includes clinical information, patient-reported-outcomes, and functional and laboratory assessment in an electronic-health-record-like environment.

A complex management solution for MS was presented by Van Hecke et al., which combines functions of an online portal for HCPs, a web/mobile application for pwMS, and an elaborated solution for brain MRI analyses [13]. For its digital biomarkers, a notable increase in sensitivity to detect disease activity was reported. To underline the market readiness and the will to translate into clinical practice, the developers acquired a CE mark and a FDA clearance.

Overall, the twelve articles in this Brain Science Special Issue demonstrated what pwMS and HCPs expect from digital innovations for the treatment of MS, what contribution these technologies can make in everyday practice, and which areas of MS assessment can benefit from these new approaches in particular. The opportunity to focus simultaneously on technical development and clinical relevance in a selected and somewhat predisposed disease, such as MS, has provided valuable insights for neurologists, epidemiologists, and developers of eHealth solutions working on chronic neurological diseases.
